# Disability weights based on patient-reported data from a multinational injury cohort

**DOI:** 10.2471/BLT.16.172155

**Published:** 2016-08-31

**Authors:** Belinda J Gabbe, Ronan A Lyons, Pamela M Simpson, Frederick P Rivara, Shanthi Ameratunga, Suzanne Polinder, Sarah Derrett, James E Harrison

**Affiliations:** a Department of Epidemiology and Preventive Medicine, Monash University, The Alfred Centre, Commercial Road, Melbourne, Victoria, 3004, Australia.; b Farr Institute, Swansea University Medical School, Swansea, Wales.; c The Harbourview Injury Prevention and Research Center, University of Washington, Seattle, United States of America.; d Section of Epidemiology and Biostatistics, University of Auckland, Auckland, New Zealand.; e Department of Public Health, Erasmus MC, Rotterdam, Netherlands.; f Injury Prevention Research Unit, University of Otago, Dunedin, New Zealand.; g Research Centre for Injury Studies, Flinders University, Adelaide, Australia.

## Abstract

**Objective:**

To create patient-based disability weights for individual injury diagnosis codes and nature-of-injury classifications, for use, as an alternative to panel-based weights, in studies on the burden of disease.

**Methods:**

Self-reported data based on the EQ-5D standardized measure of health status were collected from 29 770 participants in the Injury-VIBES injury cohort study, which covered Australia, the Netherlands, New Zealand, the United Kingdom of Great Britain and Northern Ireland and the United States of America. The data were combined to calculate new disability weights for each common injury classification and for each type of diagnosis covered by the 10th revision of the *International statistical classification of diseases and related health problems*. Weights were calculated separately for hospital admissions and presentations confined to emergency departments.

**Findings:**

There were 29 770 injury cases with at least one EQ-5D score. The mean age of the participants providing data was 51 years. Most participants were male and almost a third had road traffic injuries. The new disability weights were higher for admitted cases than for cases confined to emergency departments and higher than the corresponding weights used by the Global Burden of Disease 2013 study. Long-term disability was common in most categories of injuries.

**Conclusion:**

Injury is often a chronic disorder and burden of disease estimates should reflect this. Application of the new weights to burden studies would substantially increase estimates of disability-adjusted life-years and provide a more accurate reflection of the impact of injuries on peoples’ lives.

## Résumé

**Objectif:**

Élaborer de nouveaux coefficients de pondération de l'incapacité, fondés sur des déclarations de patients, pour les différents codes diagnostiques des traumatismes corporels et pour les différentes natures de traumatismes, en vue de les utiliser comme alternative aux pondérations fondées sur des panels dans les études sur les charges de morbidité.

**Méthodes:**

Des auto-déclarations ont été recueillies grâce au questionnaire standardisé EQ-5D d'évaluation de l'état de santé auprès de 29 770 participants de l'étude « Injury-VIBES » qui a été menée sur une cohorte de blessés en Australie, aux États-Unis d'Amérique, en Nouvelle-Zélande, aux Pays-Bas et au Royaume-Uni de Grande-Bretagne et d'Irlande du Nord. Ces données ont été combinées pour calculer de nouveaux coefficients de pondération de l'incapacité pour chaque principale catégorie de traumatisme et pour chaque type de diagnostic couvert par la 10ème révision de la Classification statistique internationale des maladies et des problèmes de santé connexes. Les coefficients de pondération ont été calculés séparément pour les patients hospitalisés et les patients uniquement pris en charge par les services d'urgence.

**Résultats:**

Il y a eu 29 770 cas de traumatismes pour lesquels au moins un score EQ-5D a été obtenu. L'âge moyen des participants ayant communiqué des données est de 51 ans. Pour la plupart, les participants étaient des hommes et près d'un tiers, des accidentés de la route. Les nouveaux coefficients de pondération sont supérieurs pour les participants hospitalisés comparativement aux patients uniquement pris en charge par des services d'urgence et supérieurs aux coefficients de pondération correspondants utilisés dans l'étude 2013 sur la charge mondiale de morbidité. Pour la plupart des catégories de traumatismes, une incapacité à long terme a été constatée.

**Conclusion:**

Un traumatisme constitue souvent un trouble chronique, et les estimations des charges de morbidité devraient refléter cet état de fait. L'application de ces nouveaux coefficients de pondération aux études sur les charges de morbidité devrait permettre de nettement améliorer les évaluations des années de vie ajustées sur l’incapacité et de refléter plus précisément l'impact des traumatismes sur la vie des individus.

## Resumen

**Objetivo:**

Crear pesos de discapacidad basados en pacientes con el fin de establecer códigos de diagnóstico de lesiones individuales y clasificaciones según la naturaleza de la lesión, para su uso en estudios sobre la carga de morbilidad, como alternativa a los pesos basados en paneles.

**Métodos:**

Se recopiló información autopresentada según la medida estandarizada EQ-5D del estado de salud de 29 770 participantes en el estudio de cohorte de lesiones Injury-VIBES, que abarcó Australia, los Países Bajos, Irlanda del Norte, Nueva Zelanda, el Reino Unido de Gran Bretaña y los Estados Unidos de América. Se combinaron los datos para calcular nuevos pesos de discapacidad para cada clasificación de lesión común y para cada tipo de diagnóstico cubierto por la décima revisión de la Clasificación Estadística Internacional de Enfermedades y Problemas Relacionados con la Salud. Se calcularon los pesos de forma separada para determinar los ingresos hospitalarios y las visitas limitadas a los servicios de urgencias.

**Resultados:**

Hubo 29 770 casos de lesiones con al menos una puntuación EQ-5D. La edad media de los participantes que ofrecieron información fue de 51 años. La mayoría de los participantes eran de sexo masculino y casi un tercio de sufrió lesiones por accidentes de tráfico. Los nuevos pesos de discapacidad fueron superiores en los casos de ingresos que en los limitados a los servicios de urgencias y mayores que los pesos correspondientes utilizados por el estudio de Carga Mundial de Morbilidad de 2013. La discapacidad a largo plazo fue común en la mayoría de las categorías de lesiones.

**Conclusión:**

Una lesión suele ser un trastorno crónico y las estimaciones de la carga de morbilidad reflejan este hecho. La aplicación de los nuevos pesos a los estudios de carga podría aumentar sustancialmente la estimación de los años de vida ajustados por discapacidad, así como ofrecer un reflejo más preciso sobre el efecto de las lesiones en la vida de las personas.

## ملخص

**الغرض:**

إنشاء أوزان العجز المستندة إلى المرضى والخاصة برموز تشخيص الإصابة الفردية وتصنيفات طبيعة الاصابة، للاستعاضة عن الأوزان الصادرة من لجان الدراسات واستخدامها في الدراسات التي تتناول مشكلة أعباء المرض.

**الطريقة:**

تم جمع البيانات المُبلّغ عنها ذاتيًا والقائمة على المقياس الموحد EQ-5D الخاص بالوضع الصحي من 29,770 مشاركًا في الدراسة الحشدية للإصابات (Injury-VIBES)، والتي تم فيها تغطية بلدان مثل أستراليا وهولندا ونيوزيلندا والمملكة المتحدة لبريطانيا العظمى وأيرلندا الشمالية والولايات المتحدة الأمريكية. ولقد تم الجمع بين البيانات لحساب أوزان العجز الجديدة لكل تصنيف شائع للإصابات ولكل نوع من التشخيص صادر عن المراجعة العاشرة للتصنيف الإحصائي الدولي للأمراض والمشاكل الصحية ذات الصلة. تم حساب الأوزان بشكل منفصل لحالات الدخول إلى المستشفيات والحضور إلى أقسام الطوارئ فقط.

**النتائج:**

كانت هناك 29,770 حالة إصابة، تضم واحدة منها على الأقل حالة إصابة بدرجة EQ-5D. وكان متوسط عمر المشاركين الذين يوفرون البيانات 51 عامًا. كان معظم المشاركين من الذكور وأصيب ما يقرب من ثلثهم بإصابات ناجمة عن حوادث الطرق. وكانت أوزان العجز الجديدة أعلى للحالات التي دخلت إلى المستشفى من الحالات التي حضرت إلى أقسام الطوارئ فقط وأعلى من الأوزان المقابلة التي تستخدمها دراسة العبء العالمي للمرض لعام 2013. وكان العجز طويل الأمد شائعًا في أغلب فئات الإصابات.

**الاستنتاج:**

غالبًا ما تكون الإصابة عبارة عن اضطراب مزمن وينبغي على التقديرات الخاصة بعبء المرض أن تعكس ذلك. وسوف يساهم تطبيق الأوزان الجديدة على دراسات العبء في تحقيق زيادة كبيرة في تقديرات سنوات العمر المصححة باحتساب مدد العجز بشكل كبير وتوفير انعكاس أكثر دقة لتأثير الإصابات على حياة الأشخاص.

## 摘要

**目的:**

旨在创建基于患者的残疾权重，用于个体损伤诊断编码和损伤性质分类，并且可以在疾病负担研究中用作基于小组的权重的备选方案。

**方法:**

搜集 29 770 位损伤——VIBES 损伤队列研究参与人员的基于 EQ-5D 标准化健康状况衡量方法的自报告数据。该研究的范围覆盖澳大利亚、大不列颠及北爱尔兰联合王国、荷兰、美国和新西兰。 对数据进行了汇总以计算出第 10 版《国际疾病与健康有关问题统计分类》中涵盖的各种常见损伤类别和各类诊断的新残疾权重。 对入院和急诊患者的权重进行了单独计算。

**结果:**

29 770 例受损伤患者在 EQ-5D 评估中至少获得了一分。 提供数据的参与者的平均年龄为 51 岁。 大多数参与者为男性，接近三分之一的参与者带有道路交通事故损伤。 入院案例的新残疾权重高于急诊案例的新残疾权重，且新残疾权重高于 2013 年全球疾病负担研究中的相应权重。 大多数损伤类别中，长期残疾较为常见。

**结论:**

损伤通常为一种慢性疾病，疾病负担评估应体现这一点。 在负担研究中运用新的权重会大幅增加残疾调整寿命年的估计值，并且能够更加精确地反映出损伤对人类生命的影响。

## Резюме

**Цель:**

Создать весовые коэффициенты инвалидности, основывающиеся на сообщениях пациентов, для кодов индивидуальных диагнозов и классификаций типов травм для использования в качестве альтернативы весовым коэффициентам, определяемым на групповом обсуждении, в исследованиях травматичности.

**Методы:**

Данные, сообщаемые пациентами в рамках стандартизированного измерения состояния здоровья EQ-5D, были получены от 29 770 участников когортного исследования травм Injury-VIBES, проводившегося на территории Австралии, Нидерландов, Новой Зеландии, Соединенного Королевства и Соединенных Штатов Америки. Полученные данные были обработаны для расчета новых весовых коэффициентов инвалидности для каждой общепринятой классификации травм и для каждого типа диагнозов, включенных в 10-ю редакцию Международной статистической классификации болезней и проблем, связанных со здоровьем. Весовые коэффициенты были рассчитаны отдельно для случаев госпитализации и случаев, ограничивающихся лечением в отделении неотложной помощи.

**Результаты:**

Исследовано 29 770 случаев травм, для которых был получен по крайней мере один балл EQ-5D. Средний возраст участников, сообщивших сведения, составил 51 год. Большинство участников были мужчинами, и почти треть получили травму в результате дорожно-транспортного происшествия. Новые весовые коэффициенты инвалидности были выше для случаев госпитализации, чем для случаев, когда лечение было ограничено неотложной медицинской помощью в соответствующих отделениях, и выше, чем соответствующие весовые коэффициенты, использованные в Глобальном исследовании заболеваемости 2013 года. Долгосрочная инвалидность была типична для большинства категорий травм.

**Вывод:**

Травма зачастую является хроническим нарушением, и это должно быть отражено в показателях заболеваемости. Применение новых весовых коэффициентов в исследованиях заболеваемости приведет к значительному увеличению значения DALY (количества лет жизни с поправкой на инвалидность) и позволит более точно отразить влияние травм на жизни людей.

## Introduction

If resource allocation and policy for the reduction of the burden of health problems are to be effective, the burden posed by injuries needs to be carefully evaluated. The disability-adjusted life-year (DALY), as used in the Global Burden of Disease (GBD) 1990, 2010 and 2013 studies,^1^^,^^2^ is based on both premature mortality – i.e. years of life lost – and years lived with disability (YLD).^3^^,^^4^ The assignment of disability weights, to represent the decrease in health associated with specific diseases or injuries, is a fundamental step in the estimation of YLD.^3^^,^^5^ Different approaches to estimating disability weights^3^ can lead to substantially different estimates of DALYs and YLD.^6^^,^^7^

In panel-based studies of health burden, a lay description –a vignette – is used to represent the health impact of the condition of interest on a hypothetical affected individual. Health professionals or representatives of the general population then give the health status of that affected individual a score, or panel-based disability weight, that ranges between zero – representing no disability or perfect health – and one – representing disability equivalent to death.^3^^,^^5^ The limitations of such a panel-based approach include the uncertain generalizability of the resultant weights to different geographical and socioeconomic contexts, the difficulty of developing vignettes to represent complex and varied health impacts and the limited focus on the time-course of any disability.^4^^,^^5^

In an alternative to the panel-based approach, self-reported data collected directly from affected individuals, using multi-attribute utility instruments – such as the EQ-5D standardized measures of health status – can be used to derive case-based disability weights.^3^ An individual’s responses to a standardized set of questions can be used to determine that individual’s generic health state and then the health states of all respondents having a particular health problem can be used to assign a disability weight to that problem. It has been suggested that such case-based disability weights should be used to quantify injury burdens.^8^^–^^10^ Two studies based on injury cohorts led to case-based weights that were larger than corresponding panel-based estimates, but both studies were limited by small sample sizes.^6^^,^^7^ The GBD 2013 study incorporated case-based weights for some injury groups but was hampered by the limited availability of case-reported data.^11^ As an adjunct or alternative to the use of panel-based weights in burden of disease studies, we used pooled patient-reported data, from six longitudinal injury-outcome studies, to create case-based weights for individual injury diagnosis codes and established nature-of-injury classifications.

## Methods

### Setting

Our investigation was based on the Validating and Improving Injury Burden Estimates Study (Injury-VIBES) cohort, which consists of participants’ data from six longitudinal studies in five countries (Table 1).^19^ The main aim of the Injury-VIBES study is to improve the measurement of non-fatal injury burden through analysis of pooled, de-identified, patient-level data. Our investigation was approved by Monash University’s Human Research Ethics Committee.

**Table 1:** Six data sets used in the estimation of new disability weights for patients with injuries

Study	Country	Inclusion criteria	Follow-up (months post-injury)	Study period	No. of participants
DIPS^12^	Netherlands	Injury cases who presented to an emergency department	2.5, 5, 9 and 24	October 2001 to December 2002	8 014
NSCOT^13^	United States of America	Cases with at least one injury with an AIS score of > 2	3 and 12	July 2001 to November 2002	3 958
POIS^14^	New Zealand	Injury cases with ACC entitlement claim	3, 12 and 24	December 2007 to June 2009	2 856
VOTOR^15^	Australia	Injury cases with orthopaedic admission of > 24 hours	6 and 12	March 2007 to March 2011	15 459
VSTR^16^^,^^17^	Australia	Injury cases with ISS of > 15 and/or with admission to ICU for > 24 hours and/or requiring urgent surgery	6, 12 and 24	March 2007 to March 2011	8 213
UKBOIS^18^	United Kingdom	Injury cases who presented to emergency department or were admitted to hospital	1, 4 and 12	September 2005 to April 2007	1 219

ACC: Accident Compensation Corporation; AIS, Abbreviated injury scale; DIPS: Dutch Injury Patient Survey; ICU: intensive care unit; ISS: injury severity score; NSCOT: National Study on Costs and Outcomes of Trauma; POIS: Prospective Outcomes of Injury Study; VOTOR: Victorian Orthopaedic Trauma Outcomes Registry; VSTR: Victorian State Trauma Registry; UKBOIS: United Kingdom Burden of Injury Study.

### Data sets

We investigated persons with injury aged at least 18 years who were included in two Australian registries – that is, the Victorian State Trauma Registry^16^^,^^17^ and the Victorian Orthopaedic Trauma Outcomes Registry^15^ –in the United Kingdom Burden of Injury Study in the United Kingdom of Great Britain and Northern Ireland,^18^ the Prospective Outcomes of Injury Study in New Zealand,^14^ the National Study on Costs and Outcomes of Trauma in the United States of America^13^ and the Dutch Injury Patient Survey in the Netherlands.^12^

### Injury classifications

When possible, weights were initially calculated for each of the four-character principal diagnosis codes listed in the 10th revision of the *International statistical classification of diseases and related health problems* (ICD-10)^20^ and then mapped to each of the 47 injury groups used in the GBD 2013 study,^11^ each of the 39 EUROCOST classification groups^21^ and each of the European Injury Data Base groupings.^22^ The ICD-10 codes for the cases from the USA were derived from the ICD-9 codes used in the data set. The Dutch data set only categorized injuries into the European Injury Data Base groupings. Although we could recategorize the Dutch patients into the injury groups used in the GBD 2013 study, we could not use the data from these patients to estimate weights for individual ICD-10 diagnosis codes.

### Disability weights

In general, the patients’ responses to the questions in the three-level EQ-5D questionnaire were used to estimate disability weights. The questionnaire is designed to record a respondent’s self-reported health status in terms of five topics: (i) anxiety/depression; (ii) mobility; (iii) pain/discomfort; (iv) self-care; and (v) usual activities. For each of these topics, a respondent is asked if they have no problems, some problems or extreme problems.^23^ The three-level EQ-5D questionnaire was used for the Australian cases from 2009 onwards and for all the injury cases included in the participating British, Dutch and New Zealand data sets. For all the other cases we considered, the recorded responses to the questions in the 12-item Short Form Health Survey^24^ had to be translated into EQ-5D responses.^24^ EQ-5D responses are used to calculate a preference score for each respondent. Such scores can range from −0.59 to 1.00. Negative values and values of zero and one indicate, respectively, respondents who have health states that are worse than death or equivalent to death and respondents who are in perfect health.^1^ Disability weights were calculated at three time points – that is at three, six and 12 months post-injury – by subtracting the EQ-5D preference scores for respondents with a particular health problem from the age- and sex-specific norms.^23^

The average EQ-5D differences at each time point were multiplied by a factor corresponding to the length of the period over which the disability weight applied and then these weighted disability averages were summed to provide an annualized or time-averaged disability weight. Thus, the calculated averages at three, six and 12 months were multiplied by 3/12, 3/12 and 6/12, respectively, with the resulting three weighted disability averages then summed together to produce a single disability weight. The nine-month outcomes from the Dutch data set were included in the 12-month estimates. Weights calculated at 12 months post-injury – hereafter called 12-month weights – were assumed to represent both the degree of residual disability at 12 months and the expected lifelong disability.^12^^,^^25^

We compared our new disability weights with the one-year Integration of European Injury Statistics weights^21^ and the long-term weights – for treated cases when weights for treated and untreated cases were given separately – of the GBD 2013 study.^11^ The former represent injured cases admitted to hospital while the latter represent cases who warrant “some form of health care in a system with full access to health care”.^1^^,^^21^ We calculated new disability weights separately for cases admitted to hospital and for other cases who only presented at emergency departments. Disability weights and corresponding 95% confidence intervals (CI) were calculated for each category that covered at least 30 cases.

## Results

Across the six data sets and three different time points we investigated, there were 29 770 injury cases with at least one EQ-5D score – 9003, 20 929 and 24 894 responses were recorded at three, six and 12 months post-injury, respectively. The mean age of the respondents was 51 years, most of them were male and almost a third of them had had road traffic injuries. The proportion of the cases from each data set that had been admitted to hospital ranged from 25% to 100% (Table 2). To save space, we have not reported weights for European Injury Data Base groupings but these are available from the corresponding author.

**Table 2:** Demographics of the patients from six injury cohorts who had an eligible EQ-5D summary score at three, six and/or 12 months post-injury

Characteristic	DIPS (*n* = 2 857)	NSCOT (*n* = 3 785)	POIS (*n* = 2 831)	VOTOR (*n* = 13 005)	VSTR (*n* = 6 845)	UKBOIS (*n* = 447)	Total (*n* = 29 770)
**Mean age in years (SD)**	50.5 (19.9)	46.6 (20.0)	41.1 (13.0)	55.7 (22.6)	48.0 (21.2)	55.0 (18.5)	50.9 (21.5)
**No. of patients (%)**							
Male	1 383 (48.4)	2 488 (65.7)	1 732 (61.2)	6 615 (50.9)	5 070 (74.1)	195 (43.6)	17 483 (58.7)
Admitted to hospital	1 525 (53.4)	3 785 (100)	699 (24.7)	13 005 (100)	6 845 (100)	198 (44.6)	26 057 (87.5)
With transport-related injury	789 (28.0)	1 716 (45.4)	326 (11.5)	3 284 (25.8)	3 319 (48.7)	58 (13.2)	9 492 (32.3)
With fall-related injury	0 (0.0)	1 292 (34.1)	695 (24.6)	7 623 (59.9)	2 108 (31.0)	0 (0.0)	11 718 (39.8)
With other injury	2 027 (72.0)	777 (20.5)	1 810 (63.9)	1 814 (14.3)	1 381 (20.3)	382 (86.8)	8 191 (27.9)

DIPS: Dutch Injury Patient Survey; NSCOT: National Study on Costs and Outcomes of Trauma; POIS: Prospective Outcomes of Injury Study; SD: standard deviation; VOTOR: Victorian Orthopaedic Trauma Outcomes Registry; VSTR: Victorian State Trauma Registry; UKBOIS: United Kingdom Burden of Injury Study.

### Case-based disability weights

#### GBD 2013 injury categories

There were insufficient case numbers to calculate new disability weights for admitted cases in 14 of the 40 nature-of-injury categories used in the GBD 2013 study (Table 3). Annualized new weights for the admitted cases sustaining one of the 26 other categories were relatively high for spinal cord injury, femoral fracture, hip fracture, pelvic fracture and lower airway burns, and relatively low for radius/ulna fractures, wrist/hand fractures and superficial injuries. For 22 injury categories, the annualized and 12-month new weights were higher –1.1-fold to 22.2-fold higher – than the corresponding GBD 2013 weights (Table 3). However, the new weights for hospitalized cases of severe traumatic brain injury and spinal cord lesion at neck level were lower than the corresponding GBD 2013 weights (Table 3).

**Table 3:** New disability weights for each of the injury categories used in the Global Burden of Disease 2013 study, as derived from the responses of patients, from six injury cohorts, who were admitted to hospital

Injury category^a^	*n*^b^	Mean new weights (95% CI)		Mean GBD 2013 long-term weights (95% CI)**^c^**
		Annualized	At 12 months post-injury	
Fracture of patella, tibia, fibula or ankle	3267	0.163 (0.154 to 0.171)	0.142 (0.132 to 0.152)	0.055 (0.036 to 0.081)
Fracture of hip	2407	0.281 (0.268 to 0.294)	0.273 (0.259 to 0.287)	0.058 (0.038 to 0.084)
Fracture of radius or ulna	2316	0.081 (0.071 to 0.091)	0.070 (0.059 to 0.081)	0.043 (0.028 to 0.064)^d^
Moderate traumatic brain injury	2310	0.197 (0.185 to 0.210)	0.186 (0.172 to 0.200)	0.231 (0.156 to 0.324)
Fracture of vertebral column	1550	0.184 (0.170 to 0.198)	0.168 (0.152 to 0.183)	0.111 (0.075 to 0.156)
Severe chest injury	1382	0.180 (0.165 to 0.195)	0.162 (0.146 to 0.178)	0.047 (0.030 to 0.070)
Fracture of clavicle, scapula or humerus	1289	0.153 (0.138 to 0.168)	0.142 (0.126 to 0.159)	0.035 (0.021 to 0.053)
Fracture of femur	1078	0.263 (0.246 to 0.280)	0.243 (0.224 to 0.262)	0.042 (0.027 to 0.063)^d^
Fracture of the sternum or ribs	1010	0.185 (0.166 to 0.203)	0.179 (0.158 to 0.199)	0.103 (0.068 to 0.145)^e^
Fracture of pelvis	906	0.205 (0.185 to 0.225)	0.194 (0.172 to 0.216)	0.182 (0.123 to 0.253)
Severe traumatic brain injury	715	0.194 (0.172 to 0.217)	0.184 (0.160 to 0.208)	0.637 (0.462 to 0.789)
Abdominal or pelvic organ injury	668	0.182 (0.162 to 0.203)	0.161 (0.138 to 0.183)	NA
Muscle and tendon injuries	551	0.108 (0.088 to 0.127)	0.089 (0.067 to 0.274)	0.008 (0.003 to 0.015)
Fracture of foot bones except ankle	477	0.179 (0.156 to 0.202)	0.168 (0.143 to 0.193)	0.026 (0.015 to 0.042)^d^
Open wounds	258	0.133 (0.100 to 0.165)	0.110 (0.075 to 0.146)	0.006 (0.002 to 0.012)^e^
Spinal cord lesion at neck level	238	0.333 (0.287 to 0.379)	0.316 (0.265 to 0.366)	0.589 (0.415 to 0.748)^f^
Spinal cord lesion below neck level	179	0.373 (0.322 to 0.424)	0.356 (0.300 to 0.411)	0.296 (0.198 to 0.414)^f^
Minor traumatic brain injury	170	0.100 (0.062 to 0.138)	0.068 (0.029 to 0.106)	0.094 (0.063 to 0.133)
Fracture of wrist and other distal part of hand	153	0.085 (0.052 to 0.117)	0.070 (0.034 to 0.106)	0.014 (0.007 to 0.025)^d^
Fracture of skull	150	0.158 (0.117 to 0.199)	0.143 (0.097 to 0.187)	0.071 (0.048 to 0.100)
Fracture of face bone	135	0.150 (0.104 to 0.196)	0.140 (0.087 to 0.194)	0.067 (0.044 to 0.097)
Superficial injury	117	0.100 (0.053 to 0.148)	0.076 (0.024 to 0.128)	NA
Dislocation of shoulder	109	0.136 (0.087 to 0.184)	0.110 (0.059 to 0.160)	0.062 (0.041 to 0.088)
Dislocation of hip	55	0.188 (0.105 to 0.270)	0.171 (0.067 to 0.274)	0.016 (0.008 to 0.028)
Burn covering ≥ 20% TBSA	55	0.176 (0.100 to 0.251)	0.156 (0.077 to 0.234)	0.135 (0.092 to 0.190)^f^
Burn covering < 20% TBSA or unspecified	54	0.131 (0.048 to 0.214)	0.110 (0.021 to 0.198)	0.016 (0.008 to 0.028)
Lower airway burns	34	0.222 (0.105 to 0.339)	0.243 (0.099 to 0.386)	0.376 (0.240 to 0.524)
Nerve injury	31	0.215 (0.140 to 0.326)	0.191 (0.078 to 0.305)	0.113 (0.076 to 0.157)
Amputation of fingers, excluding thumb	22	STS	STS	0.005 (0.002 to 0.010)
Eye injuries	18	STS	STS	0.054 (0.035 to 0.081)^e^
Amputation of one lower limb	13	STS	STS	0.039 (0.023 to 0.059)f
Dislocation of knee	12	STS	STS	0.113 (0.075 to 0.160)
Amputation of toes	10	STS	STS	0.006 (0.002 to 0.012)
Crush injury	10	STS	STS	0.132 (0.089 to 0.189)
Poisoning	7	STS	STS	0.163 (0.109 to 0.227)^f^
Amputation of one upper limb	6	STS	STS	0.039 (0.024 to 0.059)^f^
Amputation of both upper limbs	4	STS	STS	0.123 (0.081 to 0.176)^f^
Amputation of thumb	1	STS	STS	0.011 (0.005 to 0.021)
Amputation of both lower limbs	0	STS	STS	0.088 (0.057 to 0.124)^f^
Drowning or non-fatal submersion	0	STS	STS	0.247 (0.164 to 0.341)

CI: confidence interval; GBD: Global Burden of Disease; NA: not available; STS: sample too small; TBSA: total body surface area.

^a^ As used in the Global Burden of Disease 2013 study.^1^

^b^ Numbers of cases, from six injury cohorts, used in the estimation of the new weights.

^c^ As reported in the Global Burden of Disease 2013 study.^1^

^d^ For untreated cases only.

^e^ Short-term weight shown because specific long-term weight unavailable.

^f^ For treated cases only.

Long-term outcome data for injury cases not admitted to hospital were only available for 16 of the nature-of-injury categories used in the GBD 2013 study (Table 4). The new disability weights for such cases were much lower than the corresponding weights for the admitted cases and several were near zero – indicating that long-term disability is unlikely to occur (Table 4).

**Table 4:** New disability weights for each of the injury categories used in the Global Burden of Disease 2013 study, as derived from the responses of patients, from six injury cohorts, who presented at emergency department but were not admitted to hospital

Injury category**^a^**	*n***^b^**	Mean new weights (95% CI)		Mean GBD 2013 long-term weights (95% CI)**^c^**
		Annualized	At 12 months post-injury	
Muscle and tendon injuries	951	0.093 (0.081 to 0.104)	0.071 (0.058 to 0.084)	0.008 (0.003 to 0.015)
Superficial injury	226	0.056 (0.031 to 0.081)	0.035 (0.007 to 0.062)	NA
Fracture of patella, tibia, fibula or ankle	157	0.063 (0.035 to 0.091)	0.015 (−0.015 to 0.045)	0.055 (0.036 to 0.081)
Open wounds	149	−0.023 (−0.046 to −0.001)	−0.043 (−0.068 to −0.018)	0.006 (0.002 to 0.012)^d^
Fracture of foot bones except ankle	147	0.043 (0.014 to 0.073)	0.016 (−0.016 to 0.048)	0.026 (0.015 to 0.042)^e^
Fracture of wrist and other distal part of hand	142	0.035 (0.004 to 0.065)	0.005 (−0.030 to 0.040)	0.014 (0.007 to 0.025)^e^
Fracture of clavicle, scapula or humerus	139	0.023 (−0.004 to 0.050)	−0.009 (−0.038 to 0.020)	0.035 (0.021 t0 0.053)
Fracture of radius or ulna	132	0.048 (0.022 to 0.074)	0.021 (−0.010 to 0.052)	0.043 (0.028 to 0.064)^e^
Fracture of the sternum or ribs	68	−0.015 (−0.065 to 0.035)	−0.028 (−0.081 to 0.025)	0.103 (0.068 to 0.145)^d^
Moderate traumatic brain injury	64	−0.009 (−0.073 to 0.055)	−0.036 (−0.100 to 0.029)	0.231 (0.156 to 0.324)
Minor traumatic brain injury	61	0.032 (−0.016 to 0.079)	0.011 (−0.043 to 0.064)	0.094 (0.063 to 0.133)
Dislocation of shoulder	60	0.046 (0.006 to 0.085)	0.017 (−0.026 to 0.060)	0.062 (0.041 to 0.088)
Fracture of femur	42	−0.001 (−0.046 to 0.044)	−0.052 (−0.096 to −0.009)	0.042 (0.027 to 0.063)^e^
Fracture of face bone	36	−0.057 (−0.096 to −0.018)	−0.076 (−0.116 to −0.036)	0.067 (0.044 to 0.097)
Dislocation of knee	35	0.101 (0.052 to 0.149)	0.057 (0.006 to 0.109)	0.113 (0.075 to 0.160)
Fracture of vertebral column	31	0.135 (0.069 to 0.201)	0.113 (0.038 to 0.187)	0.111 (0.075 to 0.156)
Abdominal or pelvic organ injury	29	STS	STS	NA
Burn covering < 20% TBSA or unspecified	29	STS	STS	0.016 (0.008 to 0.028)
Fracture of pelvis	25	STS	STS	0.182 (0.123 to 0.253)
Eye injuries	24	STS	STS	0.054 (0.035 to 0.081)^d^
Fracture of hip	19	STS	STS	0.058 (0.038 to 0.084)
Poisoning	14	STS	STS	0.163 (0.109 to 0.227)^d^
Crush injury	12	STS	STS	0.132 (0.089 to 0.189)
Dislocation of hip	10	STS	STS	0.016 (0.008 to 0.028)
Amputation of fingers, excluding thumb	4	STS	STS	0.005 (0.002 to 0.010)
Fracture of skull	3	STS	STS	0.071 (0.048 to 0.100)
Nerve injury	3	STS	STS	0.113 (0.076 to 0.157)
Spinal cord lesion at neck level	3	STS	STS	0.589 (0.415 to 0.748)^f^
Burn covering ≥ 20% TBSA	0	STS	STS	0.135 (0.092 to 0.190)^f^
Lower airway burns	0	STS	STS	0.376 (0.240 to 0.524)
Spinal cord lesion below neck level	0	STS	STS	0.296 (0.198 to 0.414)^f^
Severe traumatic brain injury	0	STS	STS	0.637 (0.462 to 0.789)
Severe chest injury	0	STS	STS	0.047 (0.030 to 0.070)
Amputation of thumb	0	STS	STS	0.011 (0.005 to 0.021)
Amputation of one upper limb	0	STS	STS	0.039 (0.024 to 0.059)^f^
Amputation of both upper limbs	0	STS	STS	0.123 (0.081 to 0.176)^f^
Amputation of toes	0	STS	STS	0.006 (0.002 to 0.012)
Amputation of one lower limb	0	STS	STS	0.039 (0.023 to 0.059)^f^
Amputation of both lower limbs	0	STS	STS	0.088 (0.057 to 0.124)^f^
Drowning or non-fatal submersion	0	STS	STS	0.247 (0.164 to 0.341)

CI: confidence interval; GBD: Global Burden of Disease; NA: not available; STS: sample too small; TBSA: total body surface area.

^a^ As used in the global burden of disease 2013 study.^1^

^b^ Numbers of cases, from six injury cohorts, used in the estimation of the new weights.

^c^ As reported in the global burden of disease 2013 study.^1^

^d^ Short-term weight shown because long-term specific weight unavailable.

^e^ For untreated cases only.

^f^ For treated cases only.

#### EUROCOST injury groups

Annualized new disability weights were calculated for admitted cases sustaining injuries in 31 EUROCOST groups (Table 5). These new weights were lower than the corresponding Integration of European Injury Statistics weights for all but three groups – facial fractures, open facial wounds and spinal cord injuries (Table 5) – and higher than the corresponding new weights for cases not admitted to hospital, several of which were close to – or less than – zero (Table 6).

**Table 5:** New disability weights for the nature-of-injury groups used by EUROCOST, as derived from the responses of patients, from six injury cohorts, who were admitted to hospital

Nature-of-injury group**^a^**	New weights			EUROCOST weights**^b^**	
	*n*	Mean (95% CI)		*n*	Mean
		Annualized	At 12 months post-injury		
Other skull – brain injury	3173	0.195 (0.184 to 0.206)	0.184 (0.172 to 0.192)	570	0.299
Fracture of knee/lower leg	2442	0.188 (0.178 to 0.199)	0.172 (0.160 to 0.184)	628	0.382
Fracture of hip	2407	0.281 (0.268 to 0.294)	0.273 (0.259 to 0.287)	1364	0.449
Internal organ injury	2066	0.182 (0.169 to 0.194)	0.162 (0.149 to 0.175)	295	0.218
Fracture of wrist	1622	0.071 (0.059 to 0.082)	0.062 (0.049 to 0.075)	75	0.085
Fracture/dislocation/strain/sprain of vertebrae/spine	1593	0.187 (0.173 to 0.201)	0.170 (0.155 to 0.186)	329	0.342
Fracture of ankle	1195	0.150 (0.135 to 0.164)	0.128 (0.112 to 0.144)	483	0.234
Fracture of rib/sternum	1010	0.185 (0.166 to 0.203)	0.179 (0.158 to 0.199)	116	0.272
Fracture of elbow/forearm	910	0.116 (0.100 to 0.132)	0.099 (0.082 to 0.117)	313	0.192
Fracture of pelvis	906	0.205 (0.185 to 0.225)	0.194 (0.172 to 0.216)	207	0.272
Fracture of upper arm	677	0.172 (0.150 to 0.193)	0.164 (0.140 to 0.188)	483	0.210
Fracture of femur shaft	648	0.261 (0.239 to 0.283)	0.234 (0.210 to 0.257)	357	0.326
Fracture of foot/toes	477	0.179 (0.156 to 0.202)	0.168 (0.143 to 0.193)	87	0.222
Fracture of clavicle/scapula	453	0.123 (0.100 to 0.145)	0.107 (0.082 to 0.132)	233	0.292
Spinal cord injury	419	0.350 (0.316 to 0.384)	0.333 (0.296 to 0.370)	160	0.163
Other injury	387	0.196 (0.168 to 0.223)	0.171 (0.141 to 0.201)	313	0.242
Complex soft tissue injury of lower extremities	358	0.090 (0.067 to 0.113)	0.058 (0.034 to 0.082)	292	0.227
Concussion	170	0.100 (0.062 to 0.138)	0.068 (0.029 to 0.106)	606	0.119
Burns	143	0.170 (0.120 to 0.220)	0.159 (0.103 to 0.215)	62	0.214
Fracture of facial bones	141	0.147 (0.101 to 0.192)	0.136 (0.084 to 0.189)	168	0.120
Dislocation/strain/sprain of shoulder/elbow	140	0.119 (0.077 to 0.161)	0.095 (0.049 to 0.140)	23	0.064
Open wounds	134	0.091 (0.052 to 0.129)	0.076 (0.033 to 0.118)	146	0.136
Complex soft tissue injury of upper extremity	123	0.103 (0.059 to 0.148)	0.099 (0.047 to 0.151)	99	0.250
Superficial injury, including contusions	117	0.100 (0.053 to 0.148)	0.076 (0.024 to 0.128)	856	0.177
Dislocation/strain/sprain of knee	86	0.131 (0.089 to 0.173)	0.106 (0.058 to 0.155)	2	0.169
Fracture hand/fingers	78	0.044 (0.009 to 0.079)	0.031 (−0.013 to 0.076)	107	0.211
Dislocation/strain/sprain of ankle/foot	69	0.200 (0.149 to 0.251)	0.183 (0.123 to 0.244)	37	0.210
Open wound face	59	0.236 (0.154 to 0.318)	0.215 (0.122 to 0.308)	131	0.204
Dislocation/strain/sprain of hip	58	0.189 (0.111 to 0.269)	0.170 (0.072 to 0.268)	176	0.337
Open wound head	39	0.092 (0.006 to 0.178)	0.037 (−0.053 to 0.127)	171	0.224
Dislocation/strain/sprain of wrist/hand/fingers	18	STS	STS	19	0.254
Eye injury	18	STS	STS	31	0.245
Whiplash, neck sprain, distortion of cervical spine	15	STS	STS	12	0.571
Foreign body	7	STS	STS	59	0.180
Poisoning	7	STS	STS	129	0.145

CI: confidence interval; STS: sample too small.

^a^ As used by EUROCOST.^21^

^b^ Time-weighted Integration of European Injury Statistics weights for 12 months post-injury.^21^

**Table 6:** New disability weights for the nature-of injury-groups used by EUROCOST, as derived from the responses of patients, from six injury cohorts, who presented at emergency department but were not admitted to hospital

Nature-of-injury group^a^	*n*	Mean new weights (95%CI)	
		Annualized	At 12 months post-injury
Fracture/dislocation/strain/sprain of vertebrae/spine	270	0.127 (0.105 to 0.150)	0.112 (0.086 to 0.138)
Dislocation/strain/sprain of knee	241	0.093 (0.073 to 0.114)	0.062 (0.039 to 0.085)
Superficial injury, including contusions	228	0.056 (0.031 to 0.081)	0.034 (0.007 to 0.062)
Dislocation/strain/sprain of ankle/foot	169	0.070 (0.045 to 0.096)	0.045 (0.016 to 0.074)
Fracture of foot/toes	147	0.043 (0.014 to 0.073)	0.016 (−0.016 to 0.048)
Fracture of wrist	131	0.053 (0.025 to 0.082)	0.021 (−0.014 to 0.056)
Dislocation/strain/sprain of shoulder/elbow	119	0.075 (0.047 to 0.103)	0.054 (0.024 to 0.084)
Open wounds	114	−0.015 (−0.041 to 0.011)	−0.032 (−0.062 to −0.002)
Complex soft tissue injury of lower extremities	106	0.043 (0.012 to 0.074)	0.003 (−0.034 to 0.039)
Fracture ankle	91	0.077 (0.035 to 0.119)	0.034 (−0.013 to 0.080)
Complex soft tissue injury of upper extremity	88	0.072 (0.031 to 0.113)	0.062 (0.011 to 0.113)
Fracture of hand/fingers	83	0.037 (−0.004 to 0.078)	0.020 (−0.027 to 0.067)
Dislocation/strain/sprain of wrist/hand/fingers	82	0.044 (0.003 to 0.085)	0.010 (−0.036 to 0.056)
Other injury	69	0.035 (−0.006 to 0.076)	0.013 (−0.036 to 0.062)
Fracture of rib/sternum	68	−0.015 (−0.065 to 0.035)	−0.028 (−0.081 to 0.025)
Other skull – brain injury	67	−0.006 (−0.067 to 0.054)	−0.032 (−0.094 to 0.030)
Fracture of knee/lower leg	66	0.045 (0.012 to 0.074)	−0.011 (−0.044 to 0.023)
Fracture of clavicle/scapula	63	−0.003 (−0.036 to 0.030)	−0.029 (−0.063 to 0.004)
Fracture of elbow/forearm	62	0.020 (−0.016 to 0.057)	−0.014 (−0.053 to 0.025)
Concussion	61	0.032 (−0.016 to 0.079)	0.011 (−0.043 to 0.064)
Fracture of upper arm	50	0.081 (0.033 to 0.129)	0.036 (−0.014 to 0.086)
Whiplash, neck sprain, distortion of cervical spine	41	0.111 (0.048 to 0.174)	0.093 (0.020 to 0.165)
Fracture of facial bones	36	−0.057 (−0.096 to −0.018)	−0.076 (−0.116 to −0.036)
Internal organ injury	29	STS	STS
Burns	29	STS	STS
Fracture of pelvis	25	STS	STS
Eye injury	24	STS	STS
Dislocation/strain/sprain of hip	24	STS	STS
Open wound on head	23	STS	STS
Fracture of hip	19	STS	STS
Open wound on face	15	STS	STS
Poisoning	14	STS	STS
Foreign body	10	STS	STS
Fracture of femur shaft	4	STS	STS
Spinal cord injury	3	STS	STS

CI: confidence interval; STS: sample too small.

^a^ As used by EUROCOST.^21^

#### ICD-10 diagnosis codes

Within the data sets we investigated, there were at least 30 cases admitted to hospital for each of 80 ICD-10 codes (Table 7; available at: http://www.who.int/bulletin/volumes/94/10/16-172155) and at least 30 cases who only presented in an emergency department for each of 16 ICD-10 codes (Table 8; available at: http://www.who.int/bulletin/volumes/94/10/16-172155). The new weights for most intracranial injuries were similar but those for skull fracture codes and concussion were relatively low. The new disability weights for individual ICD-10 codes indicated wide variation in fracture-related disability within body regions. For example, the new weight for lateral malleolus fractures was substantially lower than the new weights for other fractures in the knee or lower leg (Table 7; available at: http://www.who.int/bulletin/volumes/94/10/16-172155).

**Table 7:** New disability weights for the primary diagnosis codes of the International Statistical Classification of Diseases (ICD), as derived from the responses of patients, from six injury cohorts, who were admitted to hospital

Body region, code^a^	Diagnosis	*n*	Mean new weights (95%CI)	
			Annualized	At 12 months post-injury
**Head**				
S02.1	Fracture of base of skull	92	0.149 (0.100 to 0.197)	0.139 (0.085 to 0.194)
S02.4	Fracture of malar and maxillary bones	49	0.195 (0.119 to 0.270)	0.182 (0.095 to 0.270)
S06.0	Concussion	108	0.147 (0.100 to 0.193)	0.121 (0.072 to 0.169)
S06.1	Traumatic cerebral oedema	79	0.276 (0.197 to 0.354)	0.257 (0.177 to 0.338)
S06.2	Diffuse brain injury	466	0.205 (0.177 to 0.234)	0.197 (0.166 to 0.227)
S06.3	Focal brain injury	483	0.169 (0.143 to 0.194)	0.158 (0.131 to 0.186)
S06.4	Epidural haemorrhage	281	0.185 (0.153 to 0.217)	0.161 (0.127 to 0.196)
S06.5	Traumatic subdural haemmorhage	783	0.210 (0.187 to 0.234)	0.203 (0.178 to 0.227)
S06.6	Traumatic subarachnoid haemmorhage	597	0.214 (0.188 to 0.241)	0.206 (0.177 to 0.234)
S06.8	Other intracranial injuries	249	0.174 (0.139 to 0.209)	0.160 (0.122 to 0.197)
S09.9	Unspecified injury of head	59	0.239 (0.165 to 0.313)	0.212 (0.128 to 0.297)
**Neck**				
S12.0	Fracture of first cervical vertebra	49	0.129 (0.054 to 0.205)	0.104 (0.025 to 0.183)
S12.1	Fracture of second cervical vertebra	179	0.183 (0.137 to 0.230)	0.186 (0.132 to 0.241)
S12.2	Fracture of other cervical vertebra	319	0.146 (0.117 to 0.175)	0.133 (0.101 to 0.166)
S14.0	Concussion and oedema of cervical spinal cord	36	0.235 (0.128 to 0.342)	0.241 (0.112 to 0.370)
S14.1	Other and unspecified injuries of cervical spinal cord	199	0.347 (0.297 to 0.396)	0.324 (0.270 to 0.378)
**Thorax**				
S22.0	Fracture thoracic vertebra	351	0.207 (0.176 to 0.238)	0.194 (0.161 to 0.228)
S22.4	Multiple fractures of ribs	866	0.187 (0.167 to 0.207)	0.183 (0.160 to 0.205)
S22.5	Flail chest	61	0.211 (0.132 to 0.290)	0.180 (0.098 to 0.262)
S24.1	Other and unspecified injuries of thoracic spinal cord	106	0.435 (0.367 to 0.502)	0.403 (0.331 to 0.475)
S26.8	Other injuries of heart	96	0.142 (0.099 to 0.184)	0.127 (0.075 to 0.179)
S27.0	Traumatic pneumothorax	416	0.164 (0.137 to 0.192)	0.154 (0.124 to 0.183)
S27.1	Traumatic haemothorax	63	0.143 (0.083 to 0.202)	0.108 (0.044 to 0.172)
S27.2	Traumatic haemopneumothorax	167	0.155 (0.113 to 0.196)	0.130 (0.087 to 0.172)
S27.3	Other injuries of lung	488	0.205 (0.179 to 0.231)	0.182 (0.155 to 0.209)
S27.8	Injury of other unspecified intrathoracic organs	91	0.247 (0.176 to 0.318)	0.220 (0.149 to 0.290)
**Abdomen/lower back/lumbar spine/pelvis**				
S32.0	Fracture of lumbar vertebra	383	0.207 (0.178 to 0.237)	0.187 (0.156 to 0.219)
S32.1	Fracture of sacrum	175	0.191 (0.150 to 0.232)	0.171 (0.128 to 0.214)
S32.3	Fracture of ilium	60	0.249 (0.170 to 0.327)	0.234 (0.140 to 0.327)
S32.4	Fracture of acetabulum	213	0.242 (0.200 to 0.284)	0.233 (0.186 to 0.279)
S32.5	Fracture of pubis	525	0.179 (0.154 to 0.205)	0.171 (0.143 to 0.199)
S32.8	Fracture of other or unspecified lumbar spine or pelvis	78	0.266 (0.187 to 0.345)	0.241 (0.162 to 0.320)
S34.1	Other injury of lumbar spinal cord	51	0.316 (0.221 to 0.411)	0.328 (0.216 to 0.440)
S36.0	Injury of spleen	173	0.175 (0.136 to 0.215)	0.154 (0.111 to 0.197)
S36.1	Injury of liver or gall bladder	107	0.159 (0.110 to 0.208)	0.142 (0.088 to 0.197)
S36.4	Injury of small intestine	56	0.239 (0.141 to 0.338)	0.217 (0.112 to 0.323)
S36.5	Injury of colon	46	0.210 (0.125 to 0.295)	0.177 (0.085 to 0.268)
S36.8	Injury of other intra-abdominal organ	112	0.182 (0.132 to 0.232)	0.181 (0.121 to 0.242)
S37.0	Injury of kidney	44	0.205 (0.123 to 0.287)	0.200 (0.105 to 0.295)
**Shoulder and upper arm**				
S42.0	Fracture of clavicle	307	0.103 (0.073 to 0.132)	0.092 (0.063 to 0.122)
S42.1	Fracture of scapula	102	0.150 (0.100 to 0.199)	0.127 (0.073 to 0.181)
S42.2	Fracture of upper end of humerus	511	0.178 (0.153 to 0.203)	0.175 (0.147 to 0.203)
S42.3	Fracture of shaft of humerus	146	0.160 (0.113 to 0.207)	0.141 (0.090 to 0.192)
S42.4	Fracture of lower end of humerus	141	0.158 (0.113 to 0.203)	0.151 (0.100 to 0.203)
S43.0	Dislocation of shoulder	50	0.158 (0.079 to 0.238)	0.137 (0.055 to 0.218)
S43.1	Dislocation of acromioclavicular joint	37	0.154 (0.066 to 0.243)	0.140 (0.053 to 0.227)
**Elbow and forearm**				
S52.0	Fracture upper end of ulna	252	0.103 (0.073 to 0.132)	0.082 (0.050 to 0.114)
S52.1	Fracture upper end of radius	161	0.128 (0.091 to 0.165)	0.107 (0.067 to 0.147)
S52.2	Fracture shaft of ulna	60	0.100 (0.048 to 0.152)	0.084 (0.027 to 0.142)
S52.3	Fracture shaft of radius	63	0.073 (0.023 to 0.123)	0.027 (−0.023 to 0.077)
S52.4	Fracture of shafts of both radius and ulna	92	0.132 (0.075 to 0.190)	0.131 (0.065 to 0.197)
S52.5	Fracture lower end of radius	1339	0.061 (0.048 to 0.074)	0.053 (0.038 to 0.067)
S52.6	Fracture lower ends of both radius and ulna	208	0.110 (0.077 to 0.144)	0.107 (0.068 to 0.145)
S52.8	Fracture other parts of forearm	93	0.087 (0.039 to 0.135)	0.083 (0.028 to 0.139)
**Wrist and hand**				
S62.0	Fracture of scaphoid bone	38	0.152 (0.065 to 0.239)	0.156 (0.061 to 0.250)
**Hip and thigh**				
S72.0	Fracture neck of femur	1315	0.267 (0.249 to 0.285)	0.260 (0.241 to 0.280)
S72.1	Pertrochanteric fracture	829	0.307 (0.284 to 0.330)	0.301 (0.277 to 0.326)
S72.2	Subtrochanteric fracture	187	0.279 (0.232 to 0.326)	0.267 (0.217 to 0.318)
S72.3	Fracture shaft of femur	533	0.266 (0.243 to 0.290)	0.240 (0.214 to 0.266)
S72.4	Fracture lower end of femur	370	0.292 (0.261 to 0.322)	0.293 (0.258 to 0.328)
S72.9	Fracture of femur, part unspecified	51	0.232 (0.151 to 0.313)	0.202 (0.114 to 0.290)
S76.1	Injury of quadriceps muscle and tendon	50	0.065 (0.008 to 0.121)	0.024 (−0.033 to 0.081)
**Knee and lower leg**				
S82.0	Fracture of patella	237	0.160 (0.127 to 0.192)	0.136 (0.099 to 0.172)
S82.1	Fracture of upper end of tibia	379	0.185 (0.158 to 0.211)	0.178 (0.148 to 0.209)
S82.2	Fracture of shaft of tibia	535	0.224 (0.201 to 0.247)	0.204 (0.178 to 0.230)
S82.3	Fracture of lower end of tibia	252	0.216 (0.182 to 0.251)	0.193 (0.155 to 0.232)
S82.4	Fracture of fibula alone	106	0.187 (0.137 to 0.237)	0.192 (0.132 to 0.251)
S82.5	Fracture of medial malleolus	208	0.197 (0.161 to 0.232)	0.165 (0.128 to 0.201)
S82.6	Fracture of lateral malleolus	654	0.108 (0.089 to 0.127)	0.095 (0.074 to 0.116)
S82.8	Fracture of other parts of lower leg	721	0.118 (0.101 to 0.136)	0.098 (0.079 to 0.118)
S83.5	Sprain/strain of posterior/anterior cruciate ligament	47	0.122 (0.069 to 0.176)	0.094 (0.032 to 0.157)
S86.0	Injury of Achilles tendon	177	0.054 (0.027 to 0.081)	0.030 (0.002 to 0.058)
**Ankle and foot**				
S92.0	Fracture of calcaneus	147	0.223 (0.182 to 0.265)	0.217 (0.171 to 0.263)
S92.1	Fracture of talus	41	0.193 (0.127 to 0.259)	0.167 (0.089 to 0.245)
S92.2	Fracture of other tarsal bone	38	0.166 (0.096 to 0.236)	0.154 (0.080 to 0.227)
S92.3	Fracture of metatarsal bone	132	0.177 (0.129 to 0.225)	0.173 (0.121 to 0.225)
S92.4	Fracture of great toe	57	0.091 (0.037 to 0.144)	0.094 (0.030 to 0.157)
S92.5	Fracture of other toe	38	0.163 (0.050 to 0.275)	0.140 (0.023 to 0.258)
S93.3	Dislocation of other and unspecified part of foot	32	0.277 (0.191 to 0.362)	0.252 (0.143 to 0.361)
**Multiple body regions**				
T02.3	Fracture of multiple regions of one lower limb	34	0.150 (0.081 to 0.219)	0.095 (0.022 to 0.168)

CI: confidence interval.

^a^ As used in the 10th revision of the *International statistical classification of diseases and related health problems*.^20^

**Table 8:** New disability weights for the primary diagnosis codes of the International Statistical Classification of Diseases (ICD), as derived from the responses of patients, from six injury cohorts, who presented at emergency departments but were not admitted to hospital

Body region, code^a^	Diagnosis	*n*	Mean new weights (95%CI)	
			Annualized	At 12 months post-injury
**Head**				
S06.0	Concussion	30	0.070 (0.005 to 0.134)	0.041 (−0.043 to 0.124)
**Neck**				
S13.4	Sprain and strain of cervical spine	41	0.111 (0.048 to 0.174)	0.093 (0.020 to 0.165)
**Abdomen/lower back/lumbar spine/pelvis**				
S33.5	Spain and strain of lumbar spine	163	0.109 (0.083 to 0.136)	0.097 (0.066 to 0.128)
S33.0	Traumatic rupture of lumbar intervertebral disc	40	0.174 (0.094 to 0.254)	0.147 (0.063 to 0.232)
**Shoulder and upper arm**				
S43.7	Sprain/strain of other and unspecified parts of shoulder	39	0.126 (0.072 to 0.181)	0.114 (0.061 to 0.168)
S46.0	Injury of muscle(s)/tendon(s) of the rotator cuff of shoulder	35	0.157 (0.091 to 0.223)	0.166 (0.085 to 0.248)
**Elbow and forearm**				
S52.6	Fracture lower ends of both radius and ulna	37	0.059 (0.013 to 0.104)	0.030 (−0.020 to 0.080)
S52.5	Fracture lower end of radius	35	0.085 (0.027 to 0.144)	0.072 (−0.010 to 0.154)
**Wrist and hand**				
S62.3	Fracture of other metacarpal bone	35	0.071 (−0.005 to 0.148)	0.064 (−0.024 to 0.152)
**Knee and lower leg**				
S83.2	Tear of meniscus	79	0.088 (0.052 to 0.124)	0.051 (0.013 to 0.089)
S82.6	Fracture of lateral malleolus	60	0.074 (0.025 to 0.122)	0.044 (−0.007 to 0.094)
S86.0	Injury of Achilles tendon	55	0.081 (0.037 to 0.126)	0.039 (−0.014 to 0.091)
S83.6	Sprain/strain of other and unspecified parts of knee	33	0.052 (−0.015 to 0.119)	0.023 (−0.052 to 0.099)
**Ankle and foot**				
S93.4	Sprain and strain of ankle	114	0.065 (0.034 to 0.097)	0.041 (0.005 to 0.076)
S92.3	Fracture of other tarsal bone	42	0.075 (0.017 to 0.134)	0.042 (−0.021 to 0.105)
S92.2	Fracture of metatarsal bone	31	0.062 (−0.008 to 0.131)	0.042 (−0.042 to 0.126)

CI: confidence interval.

^a^ As used in the 10th revision of the *International statistical classification of diseases and related health problems*.^20^

## Discussion

We found differences between our new weights, which were based entirely on case-reported outcomes, and the corresponding GBD 2013 weights, which were based on a combination of panel-based and case-outcome studies. It could be argued that our new weights are not directly comparable with the GBD 2013 weights, due to distinctly different approaches to weight generation, although either set of weights could be used to derive population-based measures of injury burden. The GBD studies primarily relied on the responses of a public panel or panel of experts when faced with a standardized set of brief descriptors. Our new weights are entirely based on case-reported outcomes from cohort studies in high-income countries. The GBD studies, our study and other epidemiological studies designed to generate disability weights have generally not explicitly considered the extent to which factors such as socioeconomic status, access to high-quality care, environmental barriers or resilience, adaptation and the coping strategies of injured individuals can influence the lived experience of injury-related disability.

One argument for the preferential use of panel-based weights is the potential for individuals with chronic conditions to adapt and underestimate disease burden.^26^ In general, however, our new weights – like the case-based Integration of European Injury Statistics weights – were substantially higher than the largely panel-based GBD 2013 weights. This difference was especially marked for the more common categories of injury such as fractures and dislocations. In a previous study, estimates of injury burden based on data collected from the general public were generally found to be lower than those estimated from the experiences of the injured, particularly for categories of injury that are generally perceived to be less severe, such as sprains and fractures.^6^ However, those living with spinal cord injury reported less disability than that predicted by the general public.^6^ The general public’s overestimation of the burden of disability resulting from some severe injuries may reflect the limitations of the vignette to convey the variability in disability within injuries adequately. This could explain why our new weights for severe traumatic brain injury and spinal cord lesion at neck level are substantially lower than the corresponding GBD 2013 weights. A perceived benefit of the case-based approach is the capacity to evaluate variation in disability within an injury group.

An argument for favouring estimates of disease burdens based on the perceptions of the general public over those based on the responses of the diseased has been that people living with a disease may have difficulty in placing their experiences in the context of other diseases.^26^^–^^28^ Our new weights were based on the measurement of case-reported outcomes using validated multi-attribute utility instruments. Such instruments use population preferences to create norms for health states rather than for specific conditions. Their use helps to place the experience of people living with injury into a wide context. Our new weights reflect the deviation of actual patient function from population-based norms.

The panel-based approach requires a brief lay description of what living with a particular condition is like for a typical case. The description of a typical injury case is difficult because of the potential variation in the severity of the injury and in the injury’s impact on the injured person’s life. In the GBD 2013 study, the lay description of a spinal cord lesion below neck level, as used in the GBD 2010 study, was revised to include “and no urine and bowel control”. This revision led to a sixfold increase in the corresponding disability weight – from 0.047 to 0.296.^11^ In the case-based approach, the problems associated with the variable scope and specificity of lay descriptions are avoided.

The results of our analysis indicated that all categories of injury treated via hospital admission – and most categories of injury treated only in emergency departments – were associated with persistent measurable disability. They also provided evidence of long-term disability for several injury groups where specific long-term weights were not provided by the GBD 2013 study. Similarly, where the GBD 2013 study provided long-term weights only for so-called untreated cases – for example for cases of fracture of the femur, radius or ulna – the corresponding new weights were relatively high, even though the new weights were based on cases recruited directly from health-care services in high-income countries that presumably, had access to relatively well resourced treatment.

Many EUROCOST and GBD injury groups combine several types of injury. The combination of several conditions into a single group – for which a single weight is estimated – is not problematic if the outcomes of the combined conditions are similar. Injuries of a single nature from a single body region, such as fractures within the shoulder, are often bundled together in this manner. However, our new disability weights for individual ICD-10 diagnosis codes (Table 7 and Table 8; available at: http://www.who.int/bulletin/volumes/94/10/16-172155) indicate considerable heterogeneity in disability experienced by patients with fractures in the same body region or even the same bone. For example, the new weights indicate that clavicle fractures have a much lower disability weight than fractures of the humerus or scapula and that fractures of the distal radius are less disabling than fractures of the proximal radius.

A major strength of our analysis was the large sample size – from multiple studies and health jurisdictions – which allowed weights to be estimated, for most commonly used injury classifications, for both hospital admissions and cases who were only treated in emergency departments. However, our analysis did have several limitations. The accuracy of the coding of injury diagnoses cannot be guaranteed, especially for cases attending emergency departments – whose injuries may not have been be recorded by a trained coder. Disability weights for some categories of injury were based on relatively small numbers of cases. We therefore provided 95% confidence intervals to indicate the precision of each weight estimate. Inconsistencies and errors in documentation from the GBD 2013 study^11^ sometimes made it difficult to map ICD-10 codes to the relevant GBD 2013 injury group. The six data sets we employed differed in terms of follow-up rates and availability of EQ-5D data for each time point post-injury. Responder bias may have affected the British and Dutch data sets, which showed higher losses to follow-up than the other data sets. For some data sets, there was no collection of EQ-5D scores and we needed to estimate such scores from the responses to questions in the 12-item Short Form Health Survey.

For consistency and comparability, we mapped the principal diagnosis of each case to the EUROCOST and GBD 2013 injury groups. We did not take into account additional injury diagnoses even though disability at 12 months post-injury is known to increase with the number of injuries affecting the patient.^29^ Future evaluation of injury weights should consider multiple injuries. Our method ignored recovery within three months and the data sets we used predominantly included cases of falls and road trauma. Penetrating injuries were underrepresented.

Our weights were also calculated using data from adult cases only. While the GBD studies do cover all age groups, the vignettes used in these studies have not accounted for differences between children and adults and the GBD weights have simply been assumed to be applicable to all ages. It is plausible that there are differences in the recovery trajectories of children and adults, although the magnitude of these differences is not yet known. Like the GBD 2013 weights, our new weights do not explicitly consider the presence of comorbidity. However, the new weights are calculated from responses to a multi-attribute utility instrument that included age-specific population preferences – and age is a partial proxy for comorbidity.

Our new weights were based entirely on data collected in high-income countries and it remains unclear if they could and should be applied to cases in low- and middle-income countries. Finally, we considered any disability reported 12 months post-injury as persistent. While some studies on injuries have shown little or no improvement after more than 12 months,^12^^,^^25^ others have shown such late improvement as well as nonlinear recovery trajectories.^30^^,^^31^

In conclusion, new case-based disability weights have been estimated for individual injury-related ICD-10 diagnosis codes and commonly used injury groups. In general, these weights were higher than the corresponding largely panel-based weights that have been estimated previously. Long-term disability was evident in all categories of injuries admitted to hospital. The findings indicate that injury is often a chronic disorder and burden of disease estimates should reflect this. The impact of applying the new disability weights to DALY calculations will depend on the injury incidence profile of the population studied. A similar case-based approach could be used to determine disability weights for other conditions.
